# Fcγ receptor targeting in RA

**DOI:** 10.1186/ar3572

**Published:** 2012-02-09

**Authors:** Toshiyuki Takai, Akira Nakamura, Akiko Tobinai, Shota Endo, Masanori Inui

**Affiliations:** 1 Department of Experimental Immunology, Institute of Development, Aging and Cancer, Tohoku University, Sendai 980-8575, Japan; 2 Department of Immunology, Kanazawa Medical University, Ishikawa 920-0293, Japan

## 

The activation threshold of cells in the immune system is often tuned by cell surface molecules. Among these, Fc receptors expressed on various hematopoietic cells constitute critical elements for activating or down-modulating immune responses.

IgGFc receptors (FcγRs) were originally identified as B cell surface molecules. For more than 40 years, FcγRs have continued to attract the interest of many basic researchers and clinicians due to their intriguing IgG binding ability, which provides a critical link between the humoral and cellular branches of the immune system.

Several activating-type FcγRs, which associate with homodimeric Fc receptor common γ subunits, are crucial for the onset and exacerbation of inflammatory diseases. In contrast, a unique inhibitory FcγR, FcγRIIB, plays a critical role in keeping immune cells silent. Murine models for allergic responses and autoimmune diseases including RA illustrate the indispensable roles of activating-type FcγRs and the inhibitory FcγRIIB in the initiation and suppression of inflammation, respectively [1-5].

The ultimate goals of FcγR research are to accomplish our understanding of this molecular family and to delineate novel therapeutic strategies toward the conquest of allergic and autoimmune diseases, infectious diseases, immunodeficiency, transplantation-associated immune disorders, and malignant tumors. Although many lines of evidence indicate that a part of the intravenous Ig (IVIg)-mediated anti-inflammatory effects can be attributable to the blocking of activating-type FcγRs, recent studies have pointed out an indispensable role of FcγRIIB in therapeutic benefits of IVIg in several murine models of inflammatory diseases including RA [6]. In this session, we will give a brief summary of recent knowledge on antibody biomedicine including IVIgto you, in light of exploiting FcγRs as potential therapeutic targets for various inflammatory diseases, along with the comparison withnon-FcγR-mediated mechanisms of IVIg.
